# Allergies and risk of head and neck cancer: a case–control study

**DOI:** 10.1038/s41598-024-65051-y

**Published:** 2024-07-01

**Authors:** Sepehr Fekrazad, Elham Mohebbi, Sepideh Mehravar, Mahsa Mortaja, Farzad Teymouri, Maryam Hadji, Hamideh Rashidian, Ahmad Naghibzadeh-Tahami, Nima Rezaei, Kazem Zendehdel, Mohammad Shirkhoda

**Affiliations:** 1 Department of General Surgery, Subdivision of Surgical Oncology, Cancer Institute, Tehran University of Medical Sciences, Tehran, Iran; 2 Department of Oncology, Lombardi Comprehensive Cancer Center, Georgetown University, Washington, DC USA; 3 Cancer Research Center, Cancer Institute, Tehran University of Medical Sciences, Keshavarz Boulevard, Tehran, Iran; 4 Health Sciences Unit, Faculty of Social Sciences, Tampere University, Tampere, Finland; 5 Social Determinants of Health Research Center, Institute for Futures Studies in Health, Kerman University of Medical Sciences, Kerman, Iran; 6 Department of Biostatistics and Epidemiology, Kerman University of Medical Sciences, Kerman, Iran; 7 Center for Immunodeficiencies, Children’s Medical Center, Tehran University of Medical Sciences, Tehran, Iran; 8 Department of Immunology, School of Medicine, Tehran University of Medical Sciences, Tehran, Iran; 9 Network of Immunity in Infection, Malignancy, and Autoimmunity (NIIMA), Universal Scientific Education and Research Network (USERN), Stockholm, Sweden

**Keywords:** Allergy, Head and neck cancers, Immunology, Onco-allergy, Cancer, Immunology

## Abstract

Although the relationship between allergies and cancer has been investigated extensively, the role of allergies in head and neck cancer (HNC) appears less consistent. It is unclear whether allergies can independently influence the risk of HNC in the presence of substantial environmental risk factors, including consumption of alcohol, betel quid, and cigarettes. This study aims to find this association. We examined the relationship between allergies and HNC risk in a hospital-based case–control study with 300 cases and 375 matched controls. Logistic regression models were used to estimate odds ratios (OR) and 95% confidence intervals, controlling for age, sex, tobacco smoking and opium usage history, alcohol consumption, and socioeconomic status. Our study showed a significant reduction in the risk of HNC associated with allergy symptoms after adjusting for confounders. The risk of HNC was greatly reduced among those with any type of allergy (OR 0.42, 95% CI 0.28, 0.65). The ORs were considerably reduced by 58–88% for different kinds of allergies. The risk of HNC reduction was higher in allergic women than in allergic men (71% vs. 49%). Allergies play an influential role in the risk of HNC development. Future studies investigating immune biomarkers, including cytokine profiles and genetic polymorphisms, are necessary to further delineate the relationship between allergies and HNC. Understanding the relationship between allergies and HNC may help to devise effective strategies to reduce and treat HNC.

## Introduction

Head and neck cancers (HNC) include cancers of the pharynx, larynx, salivary glands, oral cavity, and paranasal sinus. More than 850,000 cases occur annually, accounting for five percent of all cancers. Furthermore, half a million people died from HNC in 2018^1^.

Unshakable risk factors for HNC include alcohol drinking, cigarette smoking, opium use, betel-quid leaves, and Human Papillomavirus infection, particularly in oropharyngeal cancers^2–4^, Immune system reaction has been introduced as another risk factor for HNC^1,5–7^. Any studies have investigated the relationship between immune responses in the form of allergies and the risks of cancer such as pancreas cancer and prostate cancer^8–10^. An overactive immune response to certain environmental substances in patients with a high atopic tendency—those genetically able to make more immunoglobulin E (IgE) against common allergens—is more likely to lead to allergy^11–13^. IgE’s cross-linking on the surface of specific white blood cells provokes allergic diseases such as allergic rhinitis, atopic dermatitis, asthma, and other allergies such as food allergies^11^.

The adverse effect of allergies on cancer development has been explained by different hypotheses, two of which are most popular, the ‘‘prophylaxis hypothesis’’ and the ‘‘immunosurveillance hypothesis’’. In the ‘‘immunosurveillance hypothesis’’, allergies occur as an unexpected result of a hyperactive immune system that efficiently eliminates malignant cells, thus reducing the risk of cancer. On the other hand, the ‘prophylaxis hypothesis’’ proposes that allergy symptoms are triggered by the prompt exertion of toxins, microorganisms, and environmental particles, that carry or contain carcinogens. Allergy symptoms also prevent people from coming in contact with g hazardous environments^9^. In glioma and pancreatic cancers, as cancers with highly unknown environmental risk factors, allergy has been consistently proven protective^12,13^.

On the contrary, prostate cancer risk could be a significant risk factor among the allergic (Relative Risk: 2.90; 95% CI 1.26, 6.68). A plausible explanation for the elevated risk of prostate cancer might be the activation of type 2 immune responses in allergic patients, which could suppress cellular immunity and fewer lymphocytes to defend against cancerous prostate cells. Previously, a high load of lymphocyte infiltration was demonstrated in prostate tumors following androgen ablation therapy^14,15^.

The effects of allergies on HNC seem less reliable than on glioma and pancreatic cancer. In other words, it still needs to be clarified. Also, the mechanism by which allergies can independently affect the risk of HNC in the presence of known environmental risk factors such as drinking and smoking is still unclear. Furthermore, few studies have focused on the interactions between these risk factors and allergies in HNC development^16,17^. In this study, we used the questionnaire data of more than 650 people from the Iranian Opium and Cancer Study (IROPICAN), a multicenter case–control study in 10 provinces of Iran, to investigate the relationship between allergy and the incidence of HNC, along with anatomic subsites of HNC and allergic diseases like allergic rhinitis, asthma, and eczema/atopic dermatitis food allergy, and drug allergy.

## Methods and materials

Data were obtained from the IROPICAN, a large multicenter case–control study conducted in 10 focal centers. This selection of the region was due to the primary objective—the association between opium use and cancer—of the study, and the study protocol was previously published^18,19^.

### Case selection

Head and neck squamous cell carcinomas (HNSCC) patients were recruited from April 2016 to April 2019. HNSCC cases were actively confirmed by the expert of the central center of Tehran University of Medical Sciences. Cases of non-squamous cell carcinoma were excluded except nasopharyngeal adenocarcinoma, which was rare. All patients were assigned to an International Classification of Diseases (ICD-O-3) code. We included cancers of the lip (codes C00.0–C00.6, C00.8, and C00.9), oral cavity (codes C01.9, C02.0–C02.9, C03.0, C03.1, C03.9, C04.0, C04.1, C04.8, C04.9, C05.0–C05.2, C05.8, C05.9, C06.0–C06.2, C06.8, C06.8, and C06.9), salivary glands (codes C07.9, C08.0, C08.1, C08.08, and C08.9), tonsil (codes C09.0, C09.1, C09.8, and C09.9), oropharynx (codes C10.0–10.4, C10.8 and C10.9), nasopharynx (codes C11.0–C11.3, C11.8, and C11.09), hypopharynx (codes C13.0–C13.2, C13.8, and C13.9), other and ill-defined sites in the lip, oral cavity, and pharynx (codes C14.0, C14.2, and C14.8), nasal cavity and middle ear (codes C30.0 and C30.1), sinuses (codes C12.9, C31.0–C31.3, C31.8, and C31.9), larynx (codes C32.0–C32.3, C32.8, and C32.9), other and ill-defined sites (code C76.0), and head and neck cancers were overlapping or unspecified. The codes were categorized into the lip, and oral cavity, including C00–C08 and C14, pharynx codes C09–C11 and C13, larynx codes C32, and other subsites within HNSCC codes C12, C31, C32, and C76.

### Control selection

The IROPICAN is a multicenter case–control study conducted across 10 provinces of Iran from 2016 to 2020. IROPICAN successfully enrolled 3477 controls. These controls comprised the control pool from which we selected potential control subjects for our study^20^. We selected an age, sex, and residence-matched control for each case from the IROPICAN database. The data-based contained about 800 potential controls for HNSCC cases. By power estimation, 379 controls could support 80% of the power for OR 0.5. These potential controls consisted of hospital visitors who were either relatives or friends of patients admitted to non-oncology wards, or those who visited the hospital for reasons unrelated to cancer diagnosis or treatment at the time. To mitigate selection bias, we excluded emergency rooms and maternity wards from control recruitment, as their referral patterns were often influenced by the proximity of residences. For example, individuals injured in accidents were typically directed to the nearest emergency room. Additionally, the increased incidence of car collisions among drug and alcohol users further justified this exclusion. Controls were recruited from the same hospitals as the cases or from comparable referral hospitals within the catchment area. To qualify, controls also had to have no history of cancer as reported by themselves^18–22^.

### Data collection

Through face-to-face interviews, a systematized questionnaire was used by trained interviewers for both control and case groups. This questionnaire contained complete data on demographic information, history of opium, tobacco, and alcohol use, socioeconomic status, and oral health status. Detailed questionnaires and opium exposure measurements were illustrated elsewhere^18^.

Besides the primary questionnaire, another questionnaire on allergies was filled out via telephone. The allergy questionnaire included demographics and a history of allergic rhinitis, asthma, eczema/atopic dermatitis, and food and drug allergy^23^. Phone numbers were collected in the personal contact information section of the first questionnaire. The two questionnaires were linked by a unique code which the executive manager of the study only decoded. The response rate of cases and controls was 74% and 67%,exploring demographic characteristics showed no difference between participants of the study and those who refused participation (P = 0.9). All methods were carried out following Helsinki guidelines and regulations. All experimental protocols were approved by the Tehran University of Medical Sciences ethical committee. Informed consent was obtained from all subjects and/or their legal guardian(s).

### Statistical analysis

Stata, version 14 (Stata Corp, College Station, Texas) was used for all statistical analysis. Frequencies and their percentages were calculated for categorical variables. We used logistic regression models for assessing the association between allergy and HNC status (OR with a 95% confidence interval). Potential confounders were adjusted for, namely, age, gender, province, opium use, tobacco smoking, alcohol drinking, socioeconomic status (SES), and decayed, missing, and filled teeth (DMFT). Principal component analysis (PCA) was employed to assess socioeconomic status (SES). PCA is a statistical technique used to reduce the dimensionality of a dataset while retaining most of the variability in the data. In this analysis, SES was determined by combining multiple indicators into a single composite score. The indicators included years of education, which was treated as a continuous variable, and ownership of various assets, which were treated as dichotomous variables (presence or absence). The assets considered were a washing machine, freezer, personal computer, sofa, vacuum cleaner, dishwasher, split air conditioner, owned house, and owned car. By applying PCA, we were able to generate a principal component that captured the most significant variance in the SES-related data. This principal component effectively summarized the SES of each participant based on their educational attainment and asset ownership. Once the PCA was completed, the resulting scores were used to categorize SES into low and high categories. This categorization was done based on the median value of the principal component scores. Participants with scores below the median were classified as having low SES, while those with scores above the median were classified as having high SES. This method allows for a comprehensive and nuanced assessment of SES by incorporating multiple dimensions of socioeconomic factors, ensuring a robust and reliable classification.

Allergy was accounted for asthma, allergic rhinitis, eczema/atopic dermatitis, and food and drug allergies. The doctor diagnosed allergies. Analyses were conducted for HNC and all its subsites, including lip and oral cavity, pharynx, larynx, and other subsites. The P-value for multiplicative interaction between types of allergies (e.g., the interaction between asthma and eczema/atopic dermatitis) was acquired using a Wald test of the interaction coefficient in the logistic regression.

## Results

A total of 300 HNSCC cases and 379 controls were involved in this analysis. Of which 38% of cases and 12.66% of controls were opium users (P < 0.0001). Half of the patients were tobacco users, which was significantly different between both groups (53.67% vs. 30.08%; P < 0.0001). Alcohol drinking was rare in both groups (8% vs. 4.49; P = 0.056). Most subjects were of low SES and suffered from poor oral health. Most of our cases were lip and oral cavity cancer (43.3%) and laryngeal cancer (40.67%) (Table 1).

**Table 1:** Distribution of demographics and habits for head and neck squamous cell carcinoma cases and controls.

Characteristics	Case (300)	Control (379)	P-value
Gender			
Men	224 (74.67)	278 (73.35)	
Women	76 (25.33)	101 (26.65)	0.90
Age			
< 60	217 (57.26)	160 (52.63)	
≥ 60	162 (42.74)	144 (47.37)	0.23
Place of residence			
Capital city	208 (69.33)	288 (75.99)	
Non-capital city	92 (30.67)	91 (24.01)	0.02
Regular tobacco smoking*			
Yes	161 (53.67)	114 (30.08)	
No	139 (46.33)	265 (69.92)	< 0.0001
Regular alcohol user*			
Yes	24 (8)	17 (4.49)	
No	276 (92)	362 (95.51)	0.07
Regular opium user*			
Yes	114 (38)	48 (12.66)	
No	186 (62)	331 (87.34)	< 0.0001
Socioeconomic status			
Low	173 (57.67)	151 (39.84)	
High	127 (42.33)	228 (60.16)	< 0.0001
DMFT			
< 20 (Good oral health)	100 (33.33)	186 (49.08)	
≥ 20 (Poor oral health)	200 (66.67)	193 (50.92)	< 0.0001
Cancer sub-sites			
Lip and oral cavity	130 (43.33)	–	
Pharynx	33 (11)	–	
Larynx	122 (40.67)	–	
Other HNC	15 (5)	–	

*Regular opium user was defined as using opium at least once a week for at least 6 consecutive months.

*Regular tobacco smoking was defined as smoking tobacco at least once a week for at least 6 consecutive months.

*Regular alcohol drinking was defined as drinking any alcohol at least once a week for at least 6 consecutive months.

As shown in Table 2, after adjustment for age, sex, place of residence, oral health, SES, tobacco smoking, opium use, and alcohol drinking, the risk of HNSCC was significantly reduced among those with any type of allergies (OR 0.42, 95% CI 0.28, 0.65). In detail, the risk was significantly lower among those who had a history of asthma (OR 0.42, 95% CI 0.25, 0.69), allergic rhinitis (OR 0.31, 95% CI 0.13, 0.41), drug allergy (OR 0.25, 95% CI 0.07, 0.84), food allergy (OR 0.19, 95% CI 0.05, 0.66), and eczema/atopic dermatitis (OR 0.12, 95% CI 0.03, 0.41) (Table 2). The risk of HNSCC was lower in allergic women than in allergic men (71% vs. 49%) (Supplementary Table 1).

**Table 2:** The associations of allergy with head and neck squamous cell carcinoma by allergic diseases.

Characteristics	Case (300)	Control (379)	All HNSCC	
			Crude OR (95% CI)	Adjusted OR* (95% CI)
Any allergies				
No	257 (85.67)	274 (72.30)	Referent	Referent
Yes	43 (14.33)	105 (27.70)	0.43 (0.29, 0.64)	0.42 (0.28, 0.65)
Asthma				
No	269 (89.67)	310 (81.79)	Referent	Referent
Yes	31 (10.33)	69 (18.21)	0.51 (0.32, 0.81)	0.42 (0.25, 0.69)
Allergic rhinitis				
No	292 (97.33)	340 (89.71)	Referent	Referent
Yes	8 (2.67)	39 (10.29)	0.23 (0.10, 0.51)	0.31 (0.13, 0.70)
Eczema/Atopic dermatitis				
No	297 (99)	345 (91.03)	Referent	Referent
Yes	3 (1)	34 (8.97)	0.10 (0.03, 0.33)	0.12 (0.03, 0.41)
Food allergy				
No	297 (99)	355 (93.67)	Referent	Referent
Yes	3 (1)	24 (6.33)	0.14 (0.04, 0.50)	0.19 (0.05, 0.66)
Drug allergy				
No	296 (98.67)	363 (95.78)	Referent	Referent
Yes	4 (1.33)	16 (4.22)	0.30 (0.10, 0.92)	0.25 (0.07, 0.84)

*OR adjusted for age, sex, place of residence, oral health, SES, tobacco smoking, opium use, and alcohol drinking.

*Regular opium user was defined as using opium at least once a week for at least 6 consecutive months.

*Regular tobacco smoking was defined as smoking tobacco at least once a week for at least 6 consecutive months.

*Regular alcohol drinking was defined as drinking any alcohol at least once a week for at least 6 consecutive months.

We explored the relationship between allergy and the risk of HNC by subsites (Table 3). We found that the risk of developing pharyngeal cancer in people with a history of allergies was reduced by 73% compared to people without a history of allergies (OR 0.23, 95% CI 0.06, 0.80); however, the association could not be related to any types of allergic diseases such as asthma because of a small sample size of pharyngeal cancer (33 cases). We found that 60% risk of lip and oral cavity (OR 0.40, 95% CI 0.23, 0.69) and laryngeal cancer risk (OR 0.45, 95% CI 0.21, 0.94) among those suffering from any allergies vs. non-allergic participants. Regarding other HNSCC subsites, there was no significant risk reduction (OR 0.59, 95% CI 0.13, 2.61) (Table 3). A significant reduction in lip and oral cavity cancer was associated with allergic rhinitis by 75% (OR 0.25, 95% CI 0.08, 0.73), while the primary allergic disease that was protective for laryngeal cancer, asthma, was 63% (OR 0.37, 95% CI 0.16, 0.88). Our findings could not support the protective role of eczema/ atopic dermatitis, food, and drug allergies for any anatomic subsites of HNSCC (Table 3). The multiplicative interaction of rhinitis and asthma was significantly a protective factor (OR 0.19 (95% CI 0.07, 0.50). We did not detect interactions between other allergic diseases.

**Table 3:** The associations of allergy with head and neck squamous cell carcinoma by subsites.

Allergies	Lip and oral cavity (130)		Pharynx (33)		Larynx (122)		Other HNC (15)	
	Crude OR, (95% CI)	Adjusted OR*, (95% CI)	Crude OR, (95% CI)	Adjusted OR, (95% CI)	Crude OR, (95% CI)	Adjusted OR, (95% CI)	Crude OR, (95% CI)	Adjusted OR, (95% CI)
Any allergies								
Yes	0.47 (0.28, 0.80)	0.40 (0.23, 0.69)	0.26 (0.07, 0.87)	0.23 (0.06, 0.80)	0.42 (0.24, 0.73)	0.45 (0.21, 0.94)	0.65 (0.18, 2.35)	0.59 (0.13, 2.61)
No	Referent	Referent	Referent	Referent	Referent	Referent	Referent	Referent
Asthma								
Yes	0.54 (0.29, 1.00)	0.46 (0.24, 0.88)	0.28 (0.06, 1.24)	0.22 (0.05, 1.02)	0.49 (0.25, 0.93)	0.37 (0.16, 0.88)	1.12 (0.30, 4.08)	0.83 (0.18, 3.78)
No	Referent	Referent	Referent	Referent	Referent	Referent	Referent	Referent
Allergic rhinitis								
Yes	0.27 (0.09, 0.79)	0.25 (0.08, 0.73)	0.56 (0.12, 2.44)	0.71 (0.15, 3.23)	0.14 (0.03, 0.61)	0.37 (0.07, 1.81)	–	–
No	Referent	Referent	Referent	Referent	Referent	Referent	Referent	Referent
Eczema/Atopic dermatitis								
Yes	0.54 (0.18, 1.60)	0.99 (0.16, 1.54)	0.53 (0.06, 4.09)	0.51 (0.05, 4.53)	0.14 (0.01, 1.05)	0.46 (0.04, 4.70)	–	–
No	Referent	Referent	Referent	Referent	Referent	Referent	Referent	Referent
Food allergy								
Yes	0.34 (0.10, 1.18)	0.32 (0.09, 1.13)	–	–	–	–	–	–
No	Referent	Referent	Referent	Referent	Referent	Referent	Referent	Referent
Drug allergy								
Yes	–	–	–	–	0.76 (0.25, 2.34)	1.19 (0.25, 5.57)	–	–
No	Referent	Referent	Referent	Referent	Referent	Referent	Referent	Referent

*OR adjusted for age, sex, place of residence, oral health, SES, tobacco smoking, opium use, alcohol drinking.

## Discussion

Our study revealed a significant reduction in the risk of head and neck cancer (HNC) associated with allergy symptoms, with asthma, allergic rhinitis, drug allergy, and food allergy showing particularly noteworthy risk reductions. Interestingly, this protective effect was more pronounced in women than in men, and pharyngeal cancer risk notably declined in patients with a history of allergies. However, the specific type of allergic disease could not be determined due to a low case number. On the other hand, risks of lip and oral cavity cancers decreased significantly, primarily attributed to allergic rhinitis. Conversely, no protective interaction was observed between other types of allergies, such as eczema and atopic dermatitis, and specific subsites of HNSCC. Notably, the amplified connection between allergic rhinitis and asthma emerged as a significant protective factor, while asthma also notably reduced the risk of laryngeal cancer.

Several studies have corroborated our findings regarding the inverse association between cancers and allergies. For instance, a notable meta-analysis done by Hsiao et al.^16^, an inverse association between HNC risk and allergy symptoms was demonstrated (meta-OR 0.76, 95% CI 0.63–0.91). They also found that this association was stronger in case–control studies (meta-OR 0.58, 95% CI0.44–0.78) than in cohort studies (meta-OR 0.91, 95% CI 0.71–1.17). This finding could be due to the fact that the carcinogenic process of HNC with reverse causality may influence the development of allergy symptoms, cannot be ruled out. On the other hand, in cohort studies that do not suffer from reverse causality, the main problem is the low rate of HNC development during the follow-up period to achieve sufficient statistical power and generate precise effect estimates^24^. In a large population-based case control study with more than a thousand cases conducted by Michuad et al.^17^, individuals with allergies had a 19% lower risk of oropharyngeal and laryngeal cancers but not with oral cancer regardless of gender, smoking habits, and HPV16 serostatus. It is mentioned that their inconsistence result regarding oral cancers can be due to lower power in this subset compared to others. In another case–control study conducted by Craniero et al.^25^ their results indicate a potential protective role of allergies against the development of head and neck cancer, with a notably stronger effect observed in female patients.

The inverse relationship between HNC and allergies has been explained by two main hypotheses; the ‘‘immunosurveillance hypothesis’’ and the ‘‘prophylaxis hypothesis’’. While the former attributes the protective effect to an overactive immune system detecting and eradicating malignant cells, the latter suggests that allergies aid in expelling pathogens or toxins from the body, thus preventing cancer development. Sherman et al. observed that the inverse reaction between HNC and allergies was more common in tissues that had the most contact with the external environment, such as the mouth, skin, gastrointestinal tract, etc. So, this led them to conclude that the site-specific inverse relation between particular cancers and allergies is better explained by the ‘‘prophylaxis hypothesis’’^9^.

Our case–control study also appears to be in line with the ‘‘prophylaxis hypothesis’ because the highest rates of inverse association were seen in sites with the most interference with the external environment (Lip, Oral cavity, Pharynx, and Larynx), “immunosurveillance hypotheses cannot be overlooked. Biological and immunological explanations should also be addressed for the inverse effect between allergies and cancers.

Biomarkers connecting allergies and cancers have been reviewed in only two studies^24,26^. A study comparing levels of cytokines between allergic rhinitis and HNSCC noticed no difference among their studied cytokines and interleukins (IL). The studied biomarkers were IL-2, IL-4, IL5, IL-8, IL-12, IL-13, IL-1β, IL-17, IFN-γ, TNF-α monocyte chemoattractant protein-1 (MCP)-1, macrophage inflammatory protein (MIP)-1β, granulocyte and macrophage colony-stimulating factor (GM-CSF), granulocyte and macrophage colony-stimulating factor (G-CSF), and immunity related to the T cells (IL-6, IL-7, IL-10)^24^. Another study that compared cytokine concentrations in breast cancer patients also found no differences in (IL-6, IL-1β, IFN-γ, or IL-4) between allergy and cancer groups^26^. The results of this study, together with others mentioned in the literature, showed no difference in T helper-1 and T helper-2 related cytokines ((IL-2, IL-12, IFN-γ, TNF-α) and (IL-4, IL5, IL-13), respectively) in the association between cancer and allergic developments because of type I hypersensitivity reactions. This outcome opposes epidemiological study-based theories in which differences in Th1 and Th2-related cytokines had been proposed^27–29^.

Recent studies have moved past the immune system’s Th1/Th2 theory. Subgroups of cells such as Th3, Th9, Th17, regulatory T cells, and follicular Th have demonstrated that these interactions are much more significant. Another theory relies on studies proposing that T CD4+ lymphocytes are not as inflexible as Th1 or Th2, which only produce a few cytokines^30,31^ and also facilitate the conversion of effector cells such as Th1, Th2, Th17, or regulatory T cells^32–34^. Under the influence of specific cytokines, T cell subsets can become diverse cytokine-secreting cells (34–37). Other than immune-modulating cytokines such as IL-10, IL-17, IL-23, and TGF-β (assisting tumor growth), IFN-γ, TNF-α, GM-CSF, IL-2, IL-6, IL-17, and IL-1 stimulate immune surveillance and prevent tum development^28,35^. Other mediators were also studied to define a relation between other cytokines in the connection between cancer and allergies. In a study by Lippitz et al., IL-2 (a regulatory cytokine) and TGF-β were increased. At the same time, IL-1β, IL-4, and IL-17 levels were reduced, demonstrating the immunosuppressive nature of cancer patients and a lower occurrence of allergies. These discoveries and specific cytokine levels in tumors confirm the distinction of the adverse immune response created by different cancer types^36^. In a study by Carniero et al., a moderate association between IgE and IL-17 and TGF-β levels was observed in the prostate adenocarcinoma, proposing an allergy marker with immunosuppressive reaction specified for this tumor type^25^. These findings underscore the complexity of the immune landscape in cancer and allergy interactions, shedding light on potential markers and therapeutic targets for different tumor types.

While our study provides valuable insights into the relationship between allergies and head and neck cancer, it is important to acknowledge certain limitations that may have influenced our findings. One of the limitations of our study is the lack of assessment of HPV infection. HPV is known to be a significant risk factor for certain types of head and neck cancers, particularly oropharyngeal cancers. The absence of data on HPV status in our cases and controls may have influenced our results, as the risk profile could differ between HPV-positive and HPV-negative individuals. Future studies should consider incorporating HPV status into the analysis to provide a more comprehensive understanding of the relationship between allergies and head and neck cancer risk. Furthermore, our study could not eliminate recall bias despite many cases and two-step data collection. Additionally, due to the hospital-based nature of this study, we were unable to ascertain whether the cases and controls originated from the same population.

The strengths of our study include meticulous data collection utilizing structured questionnaires administered by professional interviewers, a large sample size to mitigate selection bias and enhance statistical power, and rigorous adjustment for potential confounders such as smoking and alcohol consumption.

## Conclusion

Various mechanisms responsible for the relationship between allergies and cancers require further studies with larger sample sizes, more immune biomarkers and their relationships on a molecular level, and genetic diversity to confirm a causal association between HNC and allergy. Recognizing this association and its mechanisms could help scientists to develop effective strategies to treat and even prevent HNC by producing new immunotherapy and immune prophylaxis for this disease.

### Supplementary Information

Supplementary Table 1.

## Data Availability

The datasets used and/or analyzed during the current study are available from the corresponding author upon reasonable request.
