# Supplementary material for: Allergies and risk of head and neck cancer: a case–control study
Source: Sci Rep. 2024 Jul 1;14:15006. doi: 10.1038/s41598-024-65051-y (PMC11217420; doi:10.1038/s41598-024-65051-y)
Supplement: Supplementary file 1 — Supplementary Table 1. [file 41598_2024_65051_MOESM1_ESM.pdf]

Supplementary Table 1- The associations of allergy with head and neck squamous cell carcinoma by gender.

**Allergies**	**Men**		**Women**	
	**Crude OR (95% CI)**	**Adjusted OR* (95% CI)**	**Crude OR (95% CI)**	**Adjusted OR (95% CI)**
**Any Allergies**				
**No**	Referent	Referent	Referent	Referent
**Yes**	0.50 (0.31, 0.81)	0.51 (0.30, 0.85)	0.31 (0.15, 0.64)	0.29 (0.13, 0.64)
**Asthma**				
**No**	Referent	Referent	Referent	Referent
**Yes**	0.56 (0.32, 0.98)	0.49 (0.26, 0.90)	0.43 (0.19, 0.97)	0.39 (0.16, 0.95)
**Rhinitis**				
**Yes**	0.29 (0.11, 0.72)	0.44 (0.17, 1.14)	0.15 (0.03, 0.69)	0.07 (0.01, 0.56)
**No**	Referent	Referent	Referent	Referent
**Atopic dermatitis**				
**No**	Referent	Referent	Referent	Referent
**Yes**	0.25 (0.07, 0.90)	0.34 (0.09, 1.31)	0.55 (0.13, 2.20)	0.37 (0.07, 1.93)
**Food Allergy**				
**No**	Referent	Referent	Referent	Referent
**Yes**	0.27 (0.07, 0.98)	0.44 (0.11, 1.69)	-	-
**Drug Allergy**				
**No**	Referent	Referent	Referent	Referent
**Yes**	0.52 (0.13, 2.05)	0.42 (00.09, 1.86)	-	-

*OR adjusted for age, place of residence, oral health, SES, tobacco smoking, opium use, alcohol drinking
